# Socioeconomic and migration status as predictors of emergency caesarean section: a birth cohort study

**DOI:** 10.1186/s12884-020-2725-5

**Published:** 2020-01-13

**Authors:** C. Miani, A. Ludwig, J. Breckenkamp, O. Sauzet, I-M Doyle, C. Hoeller-Holtrichter, J. Spallek, O. Razum

**Affiliations:** 1 Department of Epidemiology and International Public Health, School of Public Health, Bielefeld University, Bielefeld, Germany; 2 Centre for Statistics (ZeSt), Bielefeld University, Bielefeld, Germany; 3 Institute for General Practice, Hannover Medical School, Hannover, Germany; 4 Department of Public Health, Institute for Health, Brandenburg University of Technology Cottbus-Senftenberg, Senftenberg, Germany

**Keywords:** Migration, Acculturation, Socioeconomic determinants, Income, Education, Caesarean section, Obstetric care, Emergency, Germany, Birth cohort

## Abstract

**Background:**

Women with a migration background are reportedly at a higher risk of emergency caesarean section. There is evidence that this is due in part to suboptimal antenatal care use and quality of care. Despite the fact that migrant women and descendants of migrants are often at risk of socioeconomic disadvantage, there is, in comparison, scarce and incomplete evidence on the role of socioeconomic position as an independent risk factor for emergency caesarean delivery. We therefore investigate whether and how migration background and two markers of socioeconomic position affect the risk of an emergency caesarean section and whether they interact with each other.

**Methods:**

In 2013–2016, we recruited women during the perinatal period in Bielefeld, Germany, collecting data on health and socioeconomic and migration background, as well as routine perinatal data. We studied associations between migration background (1st generation migrant, 2nd/3rd generation woman, no migration background), socioeconomic status (educational attainment and net monthly household income), and the outcome emergency caesarean section.

**Results:**

Of the 881 participants, 21% (*n* = 185) had an emergency caesarean section. Analyses showed no association between having an emergency caesarean section and migration status or education. Women in the lowest (< 800€/month) and second lowest (between 800 and 1750€/month) income categories were more likely (aOR: 1.96, CI: 1.01–3.81; and aOR: 2.36, CI: 1.27–4.40, respectively) to undergo an emergency caesarean section than women in the higher income groups.

**Conclusions:**

Migration status and education did not explain heterogeneity in mode of birth. Having a low household income, however, increased the chances of emergency caesarean section and thereby contributed towards producing health disadvantages. Awareness of these findings and measures to correct these inequalities could help to improve the quality of obstetric care.

## Background

Emergency caesarean sections are a consequence of unexpected complications during labor. They carry higher health risks for the mother and the infant than spontaneous vaginal births and elective caesarean sections [1, 2]. Antenatal and obstetric care aim to prevent or avoid such a course. Emergency caesarean sections are thus more common when antenatal or obstetric care are accessed late or inappropriately, or when there are quality issues in their provision.

Women with a migration background or from ethnic minorities experience a higher risk of emergency caesarean section [3–6] for reasons that include the aforementioned suboptimal antenatal care use and quality of care delivery, as well as limited agency in the doctor-patient relationship [7].

In most contexts, including European countries, women with a migration background also tend to be the most at risk of socioeconomic disadvantage compared to the general population [8]. Lower socioeconomic status, independently from or in addition to migration status, contributes to health inequalities and poorer health status, as shown by a large body of evidence [9]. However, there is, in comparison to other health outcomes, scarce evidence on the role of socioeconomic position as an independent risk factor for emergency caesarean section. Studies considering the socioeconomic position of participants do not necessarily distinguish between elective and emergency caesarean sections, [10–12] which may be problematic as the two procedures are the consequences of different pregnancy profiles and approaches to pregnancy care. Other studies tend to include only one measure of socioeconomic position, namely education or income, potentially missing the effect of other influencing factors. This relative lack of evidence is surprising, considering that some of the reasons why women with a migration background might be more at risk of undergoing an emergency caesarean section, may apply to women with lower income [3] or lower educational attainment [13]. In contrast, it has been shown that lower socioeconomic position may act as a barrier towards medical intervention, as a result of discriminatory clinical decision-making [14]. It remains unclear whether and how socioeconomic position impacts emergency caesarean section rates, and whether socioeconomic position interact with migration status. This applies especially to populations where migrant women and descendants of migrants are at risk of socioeconomic disadvantage.

Although Germany is a diverse country and one of the main reception countries for migrants in Europe, there has been little systematic investigation regarding differences in emergency caesarean section rates by migration status and socioeconomic status. In a recent prospective study conducted in Berlin, emergency caesarean section rates did not differ depending on migration status and level of education [15]. Income, however, was not included in the analysis.

In the BaBi birth cohort study set in Bielefeld, Germany, a third of the participating women have a migration background and data is available on educational attainment and income level [16]. This allows investigating whether and how a measure of migration background (namely migration status) and two markers of socioeconomic position (education and income) affect the risk of an emergency caesarean section and whether they interact with each other.

## Methods

This analysis is based on data collected in the BaBi (Babys in Bielefeld) birth cohort study [16]. Over a three-year period (2013–16), we recruited 977 women and conducted standardized computer-assisted personal interviews (CAPI) during pregnancy (*n* = 305) or shortly after birth (*n* = 672). Women were recruited in three hospitals and in gynecologists/midwives’ offices in Bielefeld, North Rhine-Westphalia, Germany. After obtaining informed written consent, we offered to conduct interviews in German, Turkish or English. Women who were not able to or comfortable answering questions in one of those three languages were excluded from the study. Women had to be at least 18 years old at the time of interview.

Following another informed written consent, we linked data from the CAPIs to routinely collected perinatal data obtained from the hospitals and midwifes. The perinatal data contains standardized quality-assurance information on pregnancy and the expectant mother’s health, and on labor, mode of delivery and birth outcomes for the mother and the child. Perinatal data was available for 908 women. Forty-one women did not give their consent to access their perinatal data and 28 records could not be retrieved. Reasons for missing records included women having given birth outside Bielefeld or technical issues. Of the 908 women for whom perinatal data was available, 881 were included in the analyses. Twenty-seven women had missing values in some of the variables of interest.

Informed written consent from all the participants was obtained for the interviews and access to their medical records. The study was approved by the ethical committee of the Medical Faculty of Muenster University and the Data Protection Board of Bielefeld University.

### Description of the main variables

#### Outcome

The outcome of interest is having, or not having, undergone an *emergency caesarean section*. The category “emergency caesarean section” refers to *unplanned (or “secondary”) caesarean sections* performed once labor has started and includes “crash” (or “urgent”) caesarean sections (also performed once labor has started, but with the mother or fetus in life-threatening condition) [17].

#### Migration background

*Migration background* was determined by country of birth [18]. Women were categorized as follows: first generation (women born abroad from parents born abroad), second generation (women born in Germany from parents both born abroad), third generation (women born in Germany from parents born in Germany, but whose first language is not German), and no migration background. In line with other studies, women born in Germany with only one parent who immigrated were considered as having no migration background [18, 19].

#### Socioeconomic determinants

*Socioeconomic status* was measured through two variables, income and education. *Income* was self-declared combined net monthly household income and classified in categories: <=800, 801–1750, 1751–2750, 2751–4000, > 4000 euro. *Education* levels were broken down in low, medium and high. The respective maximum educational attainments were (i) having completed high school, (ii) an additional vocational training/apprenticeship, (iii) a bachelor degree or equivalent and above.

### Control variables

Clinically relevant factors were also considered: *age* categories, being *a primipara (bearing a child for the first time)*, and having an *at-risk pregnancy* according to the standard antenatal care records. (i.e. presenting at least one clinically relevant risk during the pregnancy). At-risk pregnancies included for example women who experienced complications during former pregnancies, are obese or who have a serious physical (heart or lung disease, diabetes, etc.) or psychological condition.

### Analysis

We used logistic regression models first to test the associations between migration background and education and emergency caesarean section frequency, adjusting for the aforementioned clinically relevant characteristics. We then introduced income in the model, as a second measure of socioeconomic position. Because income values were missing in 81 cases, multiple imputation was used to also include these participants in the analyses. Income categories were imputed as normally distributed outcome and the imputed values were then truncated with extreme values attributed to the extreme categories. A sensitivity analysis was performed by modifying the number of imputed datasets (from 10 to 20) and modifying the imputation model. Finally, we investigated interactions between migration background and socioeconomic factors. The significance level was set at *p* < 0.05. Analyses were performed with Stata 14 (StataCorp. 2015. Stata Statistical Software: Release 14. College Station, TX: StataCorp LP).

## Results

### Description of the sample

The main characteristics of the 881 participants included in the analyses are presented in Table 1.

**Table 1:** Participants’ main characteristics, Bielefeld, Germany, 2013–16

Number of participants *n* = 881	Number	Percent
Age		
18–24	69	7.8
25–29	217	24.6
30–34	361	41.0
35–49	234	26.6
*missing values*	*0*	*0*
Risk pregnancy	405	46.0
*missing values*	0	0
Primipara	338	38.4
*missing values*	*0*	*0*
Migrant status		
1st generation	225	25.5
2nd/3rd generation	92	10.4
No migration background	564	64.0
*missing values*	*0*	*0*
Level of education		
High school	132	15.0
Vocational training/apprenticeship	292	33.2
Bachelor degree (or equivalent) and above	457	51.8
*missing values*	*0*	*0*
Net monthly household income (€)		
< = 800	75	8.5
801–1750	81	9.2
1751–2750	184	20.9
2751–4000	264	30.0
> 4000	196	22.2
*missing values*	*81*	*9.2*

Of the participants, 564 (64.0%) had no migration background, 225 (22.5%) were 1st generation migrants and 92 (10.4%) were 2nd/3rd generation women. A majority of the women with a migration background were originally from Turkey, Russia or the former Soviet Union. In terms of education, the largest group (52%) were women with a bachelor degree or above. Similarly, the majority of participants had a relatively high income, with 30% of the participants declaring a net monthly household income of 2750–4000 € and over 22% declaring a net monthly household income above 4000 €.

Descriptive statistics for the outcome of interest can be found in Table 2. Twenty-one percent of the total sample (*n* = 185) had an emergency caesarean delivery. Emergency caesarean deliveries were less frequent for women with a migration background (20% of the 1st generation migrants and 16% of the 2nd/3rd generation women vs. 22.2% of the women without migration background).

**Table 2:** Emergency caesarean sections by migration and socioeconomic status, Bielefeld, Germany, 2013–16

Emergency caesarean sections n = 185	Number	Percent
Migrant status		
1st generation	45/225	20.0
2nd & 3rd generation	15/92	16.3
No migration background	125/564	22.2
*missing values*	*0*	
Level of education		
High school	23/132	17.4
Vocational training/apprenticeship	60/292	20.5
Bachelor degree (or equivalent) and above	102/457	22.3
*missing values*	*0*	
Net monthly household income (€)		
< = 800	19/75	25.3
801–1750	21/81	25.9
1751–2750	35/184	19.0
2751–4000	46/264	17.4
> 4000	37/196	18.9
*missing values*	*27/81*	33.3

### Regression analyses

Independent variables included migration status and socioeconomic variables. Clinically relevant variables were also included in the models. For each variable, the category with the most observations was chosen as the reference. Estimates obtained using imputed data for income did not differ much from those obtained without imputed values. The models used in sensitivity analysis provided similar results.

In a first model, we used only education as a marker of socioeconomic position. Results are presented in Table 3.

**Table 3:** Chance of an emergency caesarean section, by migration status and education, adjusted for clinically relevant variables, Bielefeld, Germany, 2013–16

Main effect	Adjusted odds ratio (aOR)	95% confidence interval (CI)		*p*-value
Age groups (ref = 30–34)				
18–24	0.92	0.46	1.83	0.818
25–29	0.57	0.36	0.90	0.016
35–49	0.99	0.65	1.50	0.955
Risk pregnancy (ref = no)	1.39	0.99	1.95	0.057
Primipara (ref = no)	2.44	1.71	3.48	0.000
Migration status (ref = non migrant)				
1st generation migrant	1.08	0.72	1.61	0.930
2nd/3rd generation woman	0.78	0.42	1.43	0.422
Education level (ref = Bachelor degree (or equivalent) and above)				
High school	0.85	0.48	1.51	0.583
Vocational training/apprenticeship	1.04	0.71	1.52	0.840

Being a primipara was a predictor of having an emergency caesarean section (aOR: 2.44; CI: 1.71–3.48). Women slightly younger than the reference age category (25–29 age group compared to 30–34) were less likely to have an emergency caesarean section (aOR: 0.57; CI: 0.36–0.90). Education was not associated with the outcome.

In a second model (Table 4) we added household income as an additional marker of socioeconomic position.

**Table 4:** Chance of an emergency caesarean section, by migration status, education and income, adjusted for clinically relevant variables, Bielefeld, Germany, 2013–16

Main effect	Adjusted odds ratio (aOR)	95% confidence interval (CI)		p-value
Age groups (ref = 30–34)				
18–24	0.72	0.35	1.48	0.367
25–29	0.52	0.33	0.83	0.006
35–49	1.03	0.67	1.56	0.899
Risk pregnancy (ref = no)	1.34	0.95	1.90	0.096
Primipara (ref = no)	2.48	1.73	3.56	0.000
Migration status (ref = non-migrant)				
1st generation migrant	1.00	0.66	1.50	0.976
2nd/3rd generation woman	0.75	0.41	1.40	0.364
Education level (ref = Bachelor degree (or equivalent) and above)				
High school	0.64	0.35	1.17	0.145
Vocational training/apprenticeship	0.95	0.64	1.41	0.803
Income (ref = 2751–4000€)				
< = 800	1.96	1.01	3.81	0.047
801–1750	2.36	1.27	4.40	0.007
1751–2750	1.54	0.91	2.59	0.103
> 4000	1.02	0.62	1.67	0.941

In addition to factors already relevant in the first model, income was a predictor of emergency caesarean section in the second model. Women in the two lowest income categories were more likely (aOR: 1.96 CI: 1.00–3.81 and aOR: 2.36; CI: 1.27–4.40) to have an emergency caesarean section than women in households with an income between 2751 € and 4000 €.

The outcome of interest was not associated with the facility where women gave birth and thus not associated with particular obstetric practices in facilities possibly serving particular socioeconomic subgroups of the population (results not shown). Using non-medical antenatal services, such as pregnancy-specific gymnastics classes, in addition to standard antenatal care provided by medical personnel did not affect the difference in emergency caesarean section frequency due to income (results not shown).

In a last step, we tested for interactions between migration background and socioeconomic determinants. No significant interactions were found (results not shown).

## Discussion

### Implications of the results

We found no differences in emergency caesarean section rates across groups of women with and without migration background in the BaBi birth cohort study. This is in line with the Berlin perinatal study [15, 20]. It is important to note, though, that the BaBi participants with a migration background do not necessarily represent the diversity of migration biographies in Germany. For instance, the number of vulnerable migrants, such as recent migrants and refugees, is quite small in the study population compared to studies that showed a higher rate of emergency caesarean delivery for migrants [6, 21]. Only 8% (*n* = 18) of the women in our sample who migrated themselves had lived in Germany for 2 years or less, and 10% of the women with a migration background said they had problems communicating with their care provider during pregnancy because of their limited German language skills.

One of the striking results of our analyses is the association between income and emergency caesarean section, with the likelihood of having an emergency caesarean section being higher for the lower income groups, compared to the higher income groups. Interestingly, and again similar to the Berlin study, education was not a relevant factor in explaining differences in outcome, in both models with and without income. This underlines the importance of using more than one measure of socioeconomic position and the relevance of economic status as a determinant of the birthing process.

While the literature tends to associate higher incomes with higher elective caesarean section rates, [22–24] some authors have suggested that higher emergency caesarean section rates can be associated with lower socioeconomic status [3, 21] and point toward the inappropriateness of care and unnecessary surgical interventions among vulnerable groups. The evidence remains inconclusive though, and one can only hypothesize mechanisms that would explain the influential role of income on emergency caesarean section frequency. On the one hand, the concept of agency and a woman’s ability to make decisions or influence the care she receives, and on the other hand the attitude of care providers who can, consciously or not, accommodate to various degree the preferences of more or less affluent women [5, 25] may play a part. Evidence related to obstetric care remains limited, but a 2012 systematic review by Verlinde et al. demonstrates more generally the existence of a gradient in the doctor-patient relationship depending on the socioeconomic status of the patient and the consequences it may have on treatment options and decision-making [26]. Additional qualitative work in the context of the BaBi study that could help understand the nexus of factors at play in the patient-carer relationship and decision process would be an important contribution to the field.

It can also not be excluded that women from lower economic status may exhibit poorer prenatal health characteristics or health behaviors that were not captured in this study, as well as health-seeking behaviors that would lead to poorer birth outcomes. With regard to the latter, an analysis of the labor stage at which women present themselves at the birth facility could help test this hypothesis.

Finally, further research could also establish whether the association between income and obstetric care holds when looking at other perinatal outcomes such as episiotomy, use of uterine stimulants, analgesics in labor and anesthesia in migrant populations. Indeed, two studies conducted in Berlin [19, 27] and one in the Netherlands [28] found differences in anesthesia provision and induction of labor between women with and without migration backgrounds. While two of them included measures of socioeconomic position (education [19, 28] and neighborhood socioeconomic status [28]) none took into account the individual or household income of participants.

### Strengths and limitations

Our study has two major strengths. First, the obstetric data were collected by specialist doctors using standardized and well-established procedures, thus ensuring high data quality. Second, we collected detailed information on migration status: in Germany, there are few studies looking at health and healthcare differences between groups with different migration statuses since hospital data, registries or administrative data do not capture migration background. In addition, our recruitment strategy sought to include a high proportion of women with migration background, in an effort to avoid the common lack of inclusion of minority populations in cohort studies [29].

A limitation of our analyses is that the BaBi study sample is skewed toward more educated and wealthier women. This is partly due to the recruitment strategy which started with a passive recruitment phase during which flyers and response boxes were left in gynecologists and midwifes practices [16]. Such recruitment is more likely to attract better-educated participants than active recruitment where women are directly approached by the interviewers. With this particular socioeconomic profile, our study population is not representative of the German population. However, the distribution of education and income does not constitute a limitation in the context of our specific research question and has no consequence on the relevance of the results.

With regard to migration background, the profile of participants reflects the intention of the study, which was to include in the BaBi cohort a relatively large proportion of women originally from Turkey and of Resettlers (“*Spaetaussiedler*”), the two largest groups with a migration background in Germany [16]. As previously mentioned, the sample may be biased toward better-off migrants. We did not offer interviews in Arabic and Kurdish, at a time when a record number of refugees and migrants from the Middle East were arriving in Germany [30]. This may lead to an underestimate of the influence of migration background on the outcome. The small proportion of migrants from Sub-Saharan Africa in our cohort (about 0,6% of the sample) may reduce comparability with studies from other countries, but does not constitute a limitation in the German context, as it is similar to that in Germany as a whole [31].

## Conclusion

We found no differences in emergency caesarean section rates across groups of women with and without migration background and in relation to educational attainment. However, introducing a second measure of socioeconomic status, besides education, allowed us to show that household income was associated with the outcome, with lower income being linked to an increased chance of emergency caesarean section. In our sample, income –and income only– is a discriminating factor and contributes towards producing disadvantage in health, as well as towards explaining heterogeneity in obstetric care. Awareness of these findings and measures to correct these inequalities would help to improve the quality of obstetric care, especially for women who are at an economic disadvantage.

## Data Availability

Data are available upon request due to ethical restrictions. Interested researchers may submit requests to Dr. Céline Miani, leader of the BaBi Study, School of Public Health, Bielefeld University (Contact: Universitätsstraße 25, 33615 Bielefeld, Germany. E-mail: celine.miani@uni-bielefeld.de), or to Mrs. Anja Schmid, data protection and data security officer, Bielefeld University (Contact: Universitätsstraße 25, 33615 Bielefeld, Germany. E-mail: anja.schmid@uni-bielefeld.de).

## Abbreviations

aOR: Adjusted odds ratio
CI: Confidence interval
